# High-Grade Atrioventricular Block and Ventricular Standstill in a Pregnant Patient

**DOI:** 10.7759/cureus.98855

**Published:** 2025-12-09

**Authors:** Paul Burke, Leeona Gallagher, Timothy Witting, Devind Bhullar

**Affiliations:** 1 Internal Medicine, Armadale Hospital, Perth, AUS; 2 Cardiology, Sir Charles Gairdner Hospital, Perth, AUS; 3 Cardiology, St. John of God Subiaco Hospital, Perth, AUS

**Keywords:** atrioventricular heart block, av block in pregnancy, complete heart block in pregnancy, high-grade av block, ventricular standstill

## Abstract

Heart block during pregnancy is a rare and understudied condition that poses considerable mortality risks to both the mother and foetus. The severity of atrioventricular block (AVB) tends to worsen throughout pregnancy but may resolve postpartum in some patients, suggesting that its aetiology is linked to the haemodynamic changes of pregnancy. Management of this condition necessitates a multidisciplinary approach across cardiology, obstetrics, and anaesthetics. Pacemaker indications remain consistent with those for non-pregnant patients and can be inserted safely, particularly after the first trimester. Anaesthetists must consider the potential impacts of the Valsalva manoeuvre and neuraxial anaesthesia on maternal haemodynamics during delivery. Decisions regarding the method of delivery should be based solely on obstetric indication. This report details the case of a pregnant woman who presented with pre-syncope attributed to intermittent high-grade AVB with ventricular standstill. Transthoracic echocardiogram and cardiac magnetic resonance imaging performed during and after pregnancy, respectively, revealed a structurally normal heart. The patient was successfully treated with a leadless pacemaker, had an uncomplicated vaginal delivery and experienced no recurrence of symptoms.

## Introduction

Atrioventricular block (AVB) during pregnancy is rare, characterised by disrupted electrical coordination between the atria and ventricles [1]. AVB classifications are identified via electrocardiogram (ECG), with causes divided into acquired and congenital [2-5]. The degree of AVB tends to progressively worsen as pregnancy continues, potentially leading to poor outcomes if mismanaged [6]. Existing guidelines are based on case reports, small studies and expert opinion, yet uncertainties remain [1,7,8]. We discuss a 35-week primigravida patient with high-grade AVB and ventricular standstill, exploring the potential causes, investigations and management. Following a systematic search of PubMed, Medline, and Embase using terms “AV block,” “heart block” and “pregnancy”, we believe this is the first documented case of a leadless pacemaker used for high-grade AVB and ventricular standstill in pregnancy.

## Case presentation

A 35-week pregnant nulliparous woman in her 30s presented to her obstetrician with repeated episodes of dizziness and nausea occurring at rest. Her medical history included iron and vitamin D deficiency, corrected with medication. She had no family history of cardiac disease and no history of excessive alcohol consumption, smoking or illicit drug use. Her medications included iron polymaltose 100mg, cholecalciferol 25mcg, and multivitamins. She was referred to cardiology for pre-syncope workup; her physical examination and vital signs were normal. Initial tests, including an ECG, blood tests (detailed below) and echocardiogram were also normal. During a 24-hour period of telemetry, she experienced five distinct episodes of pre-syncope, with telemetry revealing intermittent high-grade AVB with ventricular standstill of up to seven seconds, coinciding with the timing of her symptoms. As she self-reverted to normal sinus rhythm and remained haemodynamically stable, no chronotropic drugs were utilised. Due to her rapidly approaching delivery date and unclear aetiology of her AVB, it was decided that the safest approach was to implant a leadless pacemaker.

Investigations

**Figure 1:**
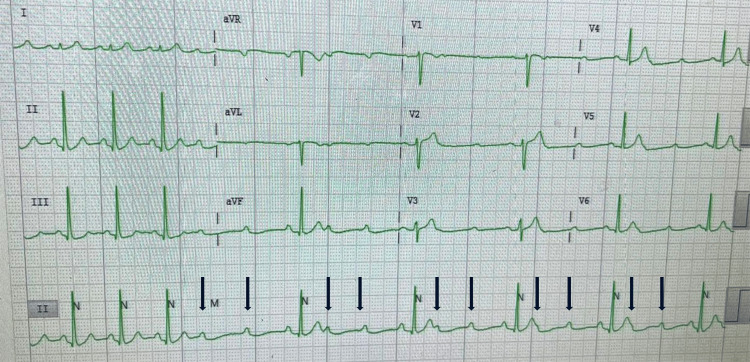
High-grade AVB with 3:1 conduction. Black arrows pointing toward consecutive non-conducted P-waves, with two non-conducted and one conducted P-wave before each QRS complex. The P-P interval remains constant, and some P-waves can be seen buried with T-waves.

**Figure 2:**
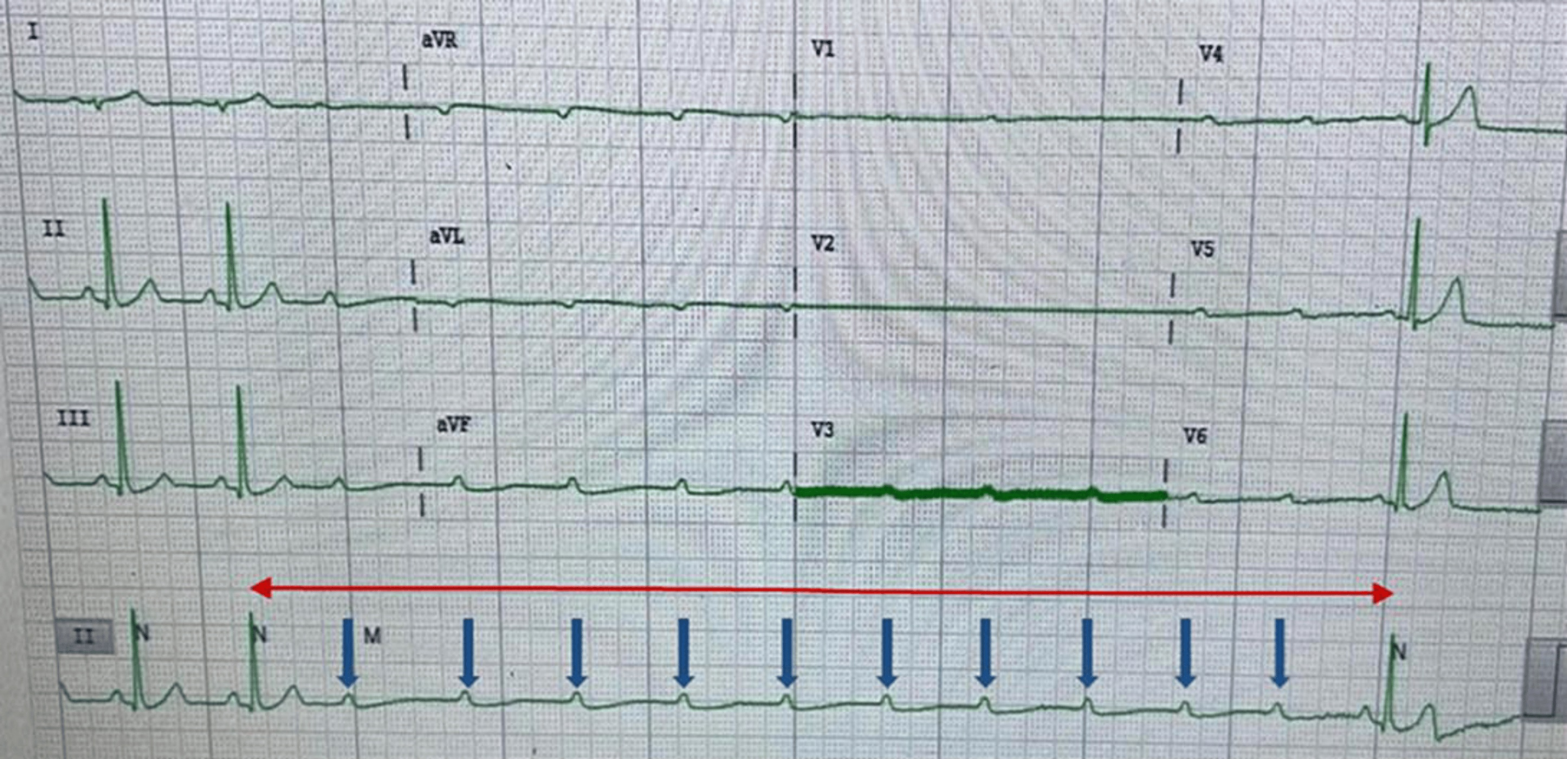
Ventricular standstill of approximately 7.68 seconds duration. Blue arrows represent non-conducted P-waves. The red arrow represents the R-R interval between ventricular depolarisations.

**Figure 3:**
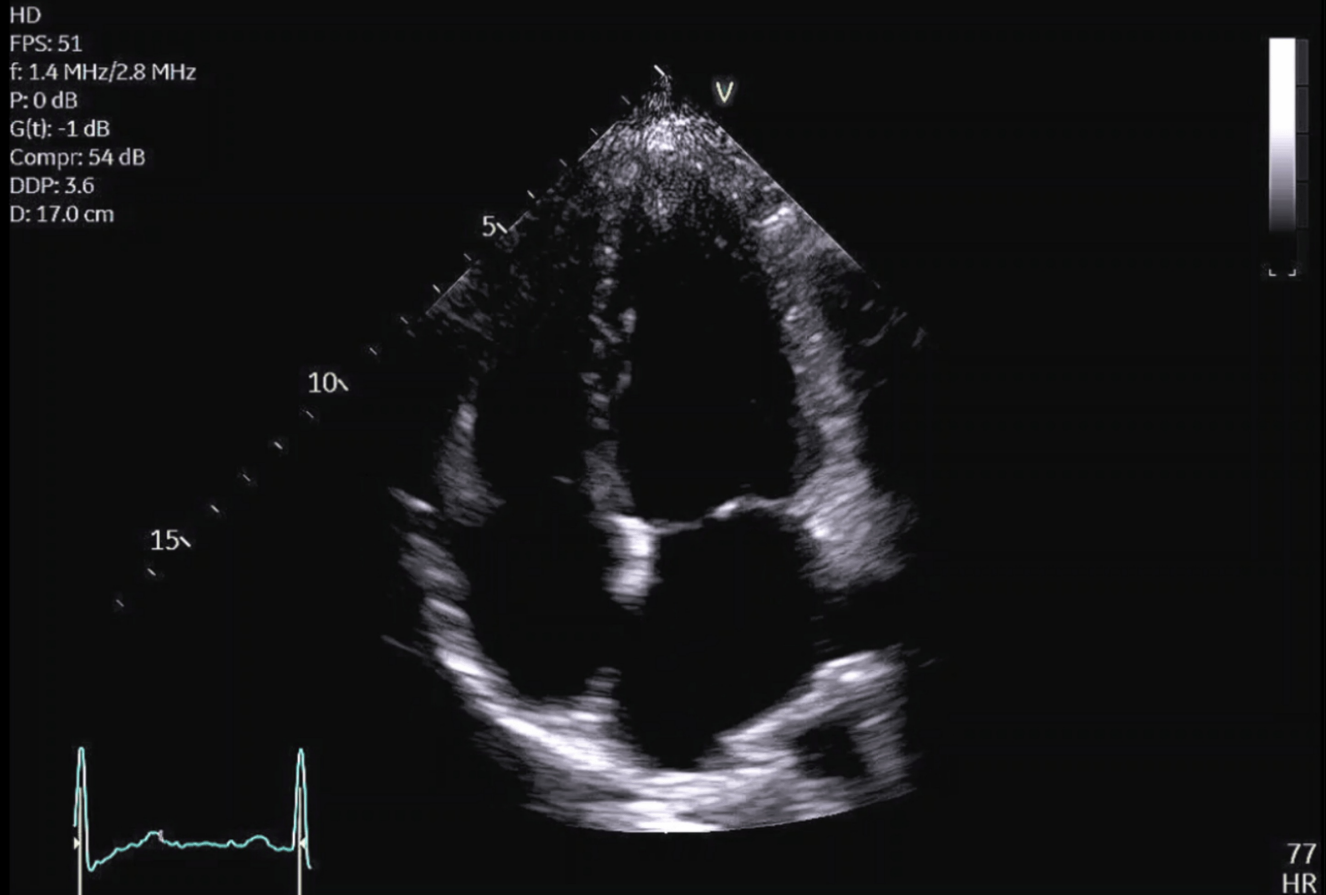
Transthoracic echocardiogram apical four-chamber view showing a structurally normal heart.

**Figure 4:**
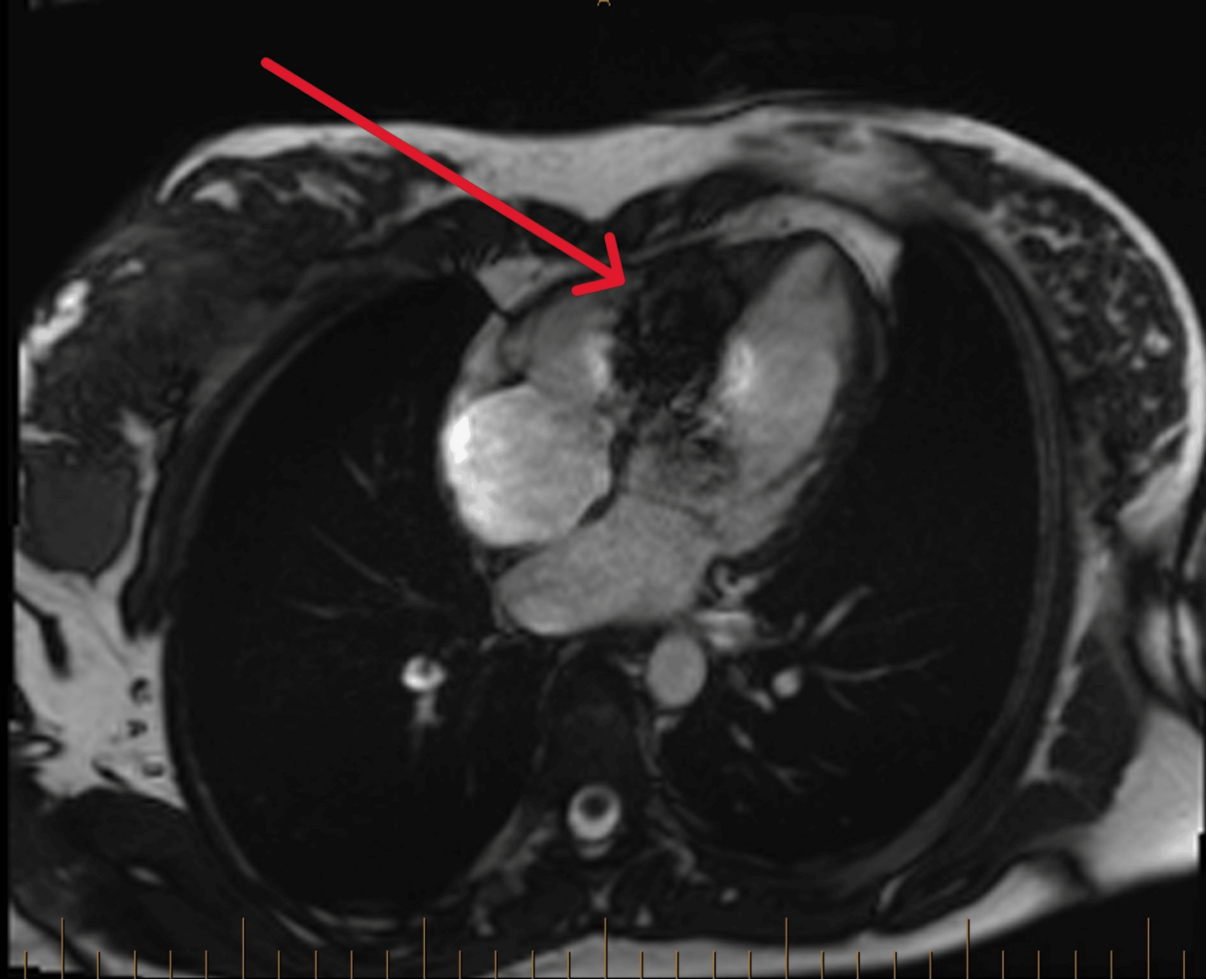
Cardiac MRI with the red arrow pointing towards the area of artefact created from the leadless pacemaker.

Serum investigations were normal and included a full blood count, electrolyte panel, thyroid-stimulating hormone (2.36 mIU/L; ref. range: 0.40-4.00mIU/L), C-reactive protein (2mg/L; ref. range: <3mg/L) and troponin (3ng/L; ref. range: <48ng/L), to check for anaemia, electrolyte abnormalities, thyroid disease and myocarditis, respectively. Initial ECGs displayed normal sinus rhythm; therefore, we opted for telemetry for a period of 24 hours, which revealed five distinct episodes of intermittent high-grade AVB with ventricular standstill of up to seven seconds, coinciding with the timing of the patient’s symptoms (Figures 1, 2). A transthoracic echocardiogram (TTE) performed during pregnancy ruled out any structural heart disease (Figure 3). Cardiac MRI was done post-delivery, avoiding the teratogenicity of contrast, which also confirmed a structurally normal heart without infiltration or fibrosis (Figure 4) [1]. However, artefact from the leadless pacemaker did preclude analysis of the mid and inferoseptum from the base to the mid ventricle.

Differential diagnosis

Reflex syncope was excluded due to the symptoms occurring at rest, absence of a history of reflex syncope and normal postural blood pressure readings.

High-grade AVB and ventricular standstill were distinguished from other bradycardias through the analysis of ECGs and telemetry.

Treatment

The patient received a leadless pacemaker, the Micra® Transcatheter Pacing System (Medtronic Inc.) via ultrasound-guided right femoral vein access at 35 weeks gestation. The pacemaker was programmed to VVI with a lower rate of 70 beats per minute (bpm). The decision to implant a leadless pacemaker was based on its reduced risk of complications, such as dislodgement, tamponade and infection compared to traditional transvenous pacemakers [9]​​​​​​. They may also be more cosmetically appealing, leaving no scar on the chest wall and offer the possibility of retrieval, if the AVB resolves postpartum [10]. 

Outcome and follow-up

The patient was discharged the day after her pacemaker insertion and check. She had a second device check and clinic review one week prior to planned delivery. She had a vaginal delivery, giving birth to a healthy baby girl at full term with no complications. Since pacemaker insertion, she has not reported any recurrence of symptoms. Her pacemaker check one week prior to delivery showed ventricular pacing at 2.7% with a lower rate at 70 bpm. Interestingly, a pacemaker check three months post delivery with the same lower rate of 70 bpm showed ventricular pacing at 19.1%. Although progressive conduction disease cannot be excluded, this is likely explained by the resolution of the physiological increase in the heart rate during pregnancy. As a result, her lower rate has been reduced to 50 bpm. She will have yearly clinic reviews with interim pacemaker checks.

## Discussion

The cardiac conduction system (CCS) consists of a specialised group of histologically distinct myocytes. Conduction myocytes are responsible for delivering sequential electrical impulses that trigger heart contraction, facilitating blood flow into the pulmonary and systemic circulation. Electrical impulses are initially generated within the sinoatrial node, located at the junction of the right atrium and superior vena cava. The impulse then propagates through the atrium until it reaches the atrioventricular node, where it is slowed down to allow adequate ventricular filling before contraction. After sufficient delay and ventricular filling, the conduction impulse travels down the interventricular septum via the His-Purkinje system, resulting in ventricular contraction. Pathology at any level of the CCS can lead to conduction disturbances and potential loss of cardiac output [11]. In our patient, the site of conduction disturbance was at the AV node, represented by the ECGs seen in Figures 1, 2.

Aetiologies of AVB can be divided into congenital and acquired causes. Autoimmune complete heart block (CHB) can develop in utero due to the passage of anti-Ro/SSA and anti-La/SSB autoantibodies, which cause fibrosis of the cardiac conduction system. Another cause is inherited progressive cardiac conduction disease, a complex condition typically occurring in individuals younger than 50 years old, without structural cardiac abnormalities and often with a positive family history. Additionally, congenital structural heart defects are another significant contributor to AVB, with L-transposition of the great arteries being the most common [3-5].

The predominant cause of acquired AVB is idiopathic sclero-degenerative disease of the conduction system, followed by both acute and chronic coronary artery diseases. Other less common causes include iatrogenic factors, cardiomyopathies, autoimmune conditions and infiltrative diseases such as Lyme carditis, amongst others [3-5].

Our patient is unlikely to be symptomatic from a sclero-degenerative or ischaemic process due to her young age. She had not undergone any cardiac procedures, nor is she on anti-arrhythmic medications. While congenital, autoimmune or infiltrative disease would be considered more likely due to her young age, her heart is structurally normal, free of fibrosis, and she has no family history of cardiac or autoimmune disease. We do acknowledge that the artefact on MRI from the leadless pacemaker precludes full assessment of the inferoseptum, and we did not specifically request serological investigations for Lyme’s disease or autoimmune antibodies. However, with a normal TTE and the absence of raised inflammatory markers and troponins, we did not think it was clinically justified, and we do not suspect there would be inflammation solely in the inferoseptum. Some studies have reported a delayed presentation of up to 30% of cases of congenital AVB, unveiled by the hyperdynamic circulation of pregnancy [12]​​​​​​. Thaman et al. theorise that AVB may be directly due to the physiological changes from pregnancy itself. They found that 71% of their untreated patients with AVB all experienced an increased severity of conduction disease as pregnancy progressed; however, these patients delivered without pacing, and their ECGs returned to baseline postpartum. They propose that the increase in pre-load due to increased circulatory volume and reduction in afterload secondary to a fall in systemic vascular resistance leads to an increase in distension of all four heart chambers. The increase in atrial stretch in particular may be enough to create a conduction disturbance or unmask one in a patient with subclinical disease [6,13].

Managing these patients requires a multidisciplinary approach involving cardiology, obstetrics, and anaesthesia. Although intricacies can arise, pacing indications are similar to the non-pregnant patient. The most recent guidelines from the Heart Rhythm Society advocate in asymptomatic haemodynamically stable patients, no intervention is required, and in those who were being considered for a permanent pacemaker (PPM) prior to pregnancy, insertion can be deferred until after delivery. For patients with irreversible symptomatic bradycardia with pre-syncope or syncope, insertion of a PPM during pregnancy is recommended. Lastly, those with symptomatic bradycardia refractory to or with contraindications to pharmacological management, a temporary pacemaker (TPM) may be used peri-partum. The European Society of Cardiology also provides guidelines on the management of this cohort of patients. Similarly, they advise that, in women with a stable narrow escape rhythm, pacing may not be needed and possibly deferred until after delivery; however, symptomatic patients or those with a wide QRS escape rhythm should have a PPM inserted during pregnancy. Radiation exposure from fluoroscopy during cardiac procedures is most significant during organogenesis in the first trimester. The foetal dosage during these procedures is unlikely to exceed 50mGy, which is considered a negligible risk for excess malignancy. To minimise radiation exposure, echocardiogram-guided insertion or electroanatomic mapping strategies may be employed [1,7,8].

A retrospective study conducted by Wang et al. examined the outcomes of pregnant women diagnosed with AVB. Eighteen patients were diagnosed with Mobitz Type 1 AVB; among them, two exhibited symptoms consistent with New York Heart Association (NYHA) Functional Classification III-IV and received temporary pacing during delivery. Of the women with Mobitz Type 2 AVB, only three developed symptoms; however, due to the potential for complications, six of the seven patients were temporarily paced during delivery. Of the 33 women with CHB, 32 received pacing and delivered without complications. The single un-paced patient required isoprenaline during labour and subsequently received a PPM post-delivery. There were no maternal or foetal deaths in this study, underscoring the benefits of pacing. Indications for pacing were based on symptoms or potential risk for complications; however, it is unclear how many of the stable asymptomatic patients would have required pacing if opted for monitoring alone [14].

Another study by Mandal et al. studied 21 women with CHB. Only one patient had a broad QRS escape rhythm; however, four women suffered Stokes-Adams attacks. Sixteen women delivered under pacing, and the remaining five patients subsequently received a pacemaker post-partum. They argue that due to the potential life-threatening risk, such as Stokes-Adams attacks, all patients with CHB should receive a PPM regardless of symptoms [15]​​​​​.

Current evidence indicates that pacing during pregnancy is safe and leads to improved outcomes for both the mother and foetus. However, there is conflicting evidence regarding which patients should receive a PPM, especially those who are haemodynamically stable but have second- or third-degree heart block.

Our patient received patient-controlled epidural anaesthesia with a solution of bupivacaine 0.0625% and fentanyl 2.5 micrograms/ml in 100 ml 0.9% normal saline at the L4/5 space. Spinal anaesthesia can cause sudden high sympathetic blockade with resultant bradycardia, which could be disastrous in the un-paced patient. To prevent this, incremental epidural top-ups or low-dose combined spinal-epidural medication is recommended. Another consideration for the anaesthetist is the risk of Valsalva-induced bradycardia, fortunately mitigated by the PPM in our patient. Though the use of chronotropic drugs in pregnancy is not well studied, they may be necessary in the unstable patient, as restoration of maternal haemodynamics is the priority to ensure the safety of both the mother and foetus [16,17].

Our patient was induced at 40 weeks and had an uncomplicated vaginal delivery, giving birth to a healthy baby girl. Induction at 40 weeks lowers the risk of emergency C-sections and stillbirth. Elective C-sections do not offer maternal benefits and lead to lower birth weight. Vaginal delivery is recommended for most women due to fewer complications. Using forceps or ventouse delivery can expedite labour, reducing maternal efforts and Valsalva effects. For patients undergoing C-sections with a PPM, bipolar diathermy or brief monopolar diathermy bursts are advised to minimise electromagnetic interference [17].

## Conclusions

This remains an understudied and fear-evoking condition for many clinicians. We suspect the aetiology in our patient's case, in the absence of structural and infiltrative heart disease, was related to the haemodynamic changes of pregnancy. However, it is difficult to ascertain whether symptoms would have recurred post-delivery due to the insertion of the pacemaker. It is essential to note that pacemaker insertion is safe during pregnancy and indications are similar to those of non-pregnant patients. Nevertheless, the asymptomatic stable patient with second or third-degree heart block remains an area of concern. There are multiple reports of these patients requiring permanent pacing in the intra- or post-partum period. Given the safety of PPM insertion and potential risks associated with untreated AVB, such as Stokes-Adams attacks, one could argue for early implantation, regardless of symptoms in this cohort. To our knowledge, following a thorough literature review, we believe this is the first documented case of a leadless pacemaker inserted for high-grade AVB and ventricular standstill during pregnancy. Leadless pacemakers are associated with a lower risk of complications and, if the AVB resolves postpartum, may present a suitable option for women considering device removal after delivery. In this instance, as our patient intends to pursue future pregnancies, the device will remain in situ, and she will have annual pacemaker checks and clinic appointments.
